# Fragment-Based Discovery of AF9 YEATS Domain Inhibitors

**DOI:** 10.3390/ijms23073893

**Published:** 2022-03-31

**Authors:** Yaqian Liu, Ruoxing Jin, Hui Lu, Kangjie Bian, Rui Wang, Lei Wang, Rui Gao, Jiahai Zhang, Jihui Wu, Xuebiao Yao, Xing Liu, Dan Liu, Xisheng Wang, Zhiyong Zhang, Ke Ruan

**Affiliations:** 1 Ministry of Education Key Laboratory for Membraneless Organelles & Cellular Dynamics, Biomedical Sciences and Health Laboratory of Anhui Province, Division of Life Sciences and Medicine, University of Science and Technology of China, Hefei 230027, China; ldd9302@mail.ustc.edu.cn (Y.L.); lucylaile@163.com (H.L.); wl0328@mail.ustc.edu.cn (L.W.); gaorui201705@163.com (R.G.); zhangjh@ustc.edu.cn (J.Z.); wujihui@ustc.edu.cn (J.W.); yaoxb@ustc.edu.cn (X.Y.); xing1017@ustc.edu.cn (X.L.); dliu919@ustc.edu.cn (D.L.); 2 Department of Chemistry, University of Science and Technology of China, Hefei 230026, China; xx520@mail.ustc.edu.cn (R.J.); kjbian@mail.ustc.edu.cn (K.B.); wangrui5@mail.ustc.edu.cn (R.W.); xswang77@ustc.edu.cn (X.W.)

**Keywords:** fragment-based lead discovery, post-translational modification, histone acylation, YEATS domain, NMR fragment-based screening

## Abstract

YEATS (YAF9, ENL, AF9, TAF14, SAS5) family proteins recognize acylated histones and in turn regulate chromatin structure, gene transcription, and stress signaling. The chromosomal translocations of ENL and mixed lineage leukemia are considered oncogenic drivers in acute myeloid leukemia and acute lymphoid leukemia. However, known ENL YEATS domain inhibitors have failed to suppress the proliferation of 60 tested cancer cell lines. Herein, we identified four hits from the NMR fragment-based screening against the AF9 YEATS domain. Ten inhibitors of new chemotypes were then designed and synthesized guided by two complex structures and affinity assays. The complex structures revealed that these inhibitors formed an extra hydrogen bond to AF9, with respect to known ENL inhibitors. Furthermore, these inhibitors demonstrated antiproliferation activities in AF9-sensitive HGC-27 cells, which recapitulated the phenotype of the CRISPR studies against AF9. Our work will provide the basis for further structured-based optimization and reignite the campaign for potent AF9 YEATS inhibitors as a precise treatment for AF9-sensitive cancers.

## 1. Introduction

Histone post-translational modification exquisitely regulates dynamic chromatin structures and gene expression [1]. Acetylation of histone lysine residues is one of the most extensively studied epigenetic markers, which are generally recognized by bromodomains and occasionally by tandem PHD domains [2,3,4]. Recently, the YEATS (YAF9, ENL, AF9, TAF14, SAS5) domains were identified as “readers” of lysine acylation, including acetylation and crotonylation. AF9 YEATS domain binds to acyl-lysine with the dissociate constants of 2.1 and 5.0 μM for crotonyl and acetyl H3K9, respectively [5,6,7,8,9]. The YEATS domain consists of approximately 120–140 residues and is evolutionarily conserved from yeast to human. Unlike the bromodomains with end-closed binding cavities, the YEATS domain adopts an end-open aromatic “sandwich” cage to recognize histone lysine acylation [5,10]. The human genome encodes four YEATS domain-containing proteins, i.e., ENL (MLLT1), YEATS2, AF9 (MLLT3), and GAS41. These four proteins are often associated with histone acetyl-transferases or chromatin remodeling complexes, implicating their roles in the regulation of chromatin structure, gene transcription, stress signaling, and DNA damage response [5,10,11,12,13,14]. Therefore, dysfunction of these YEATS domains often correlates with the onset and progression of various diseases. For instance, mutations of the AF9 YEATS domain are associated with lymphoma and glioma cancer [15,16]. AF9 and ENL are frequently fused with the mixed lineage leukemia protein as a result of chromosomal translocations. These fusion proteins are oncogenic drivers in acute myeloid leukemia and acute lymphoid leukemia [17,18,19,20]. In addition, activating ENL mutations early in renal development trigger the development of a Wilms’ tumor [21]. These studies underpin the pursuit of small-molecule inhibitors against the AF9/ENL YEATS domain.

The small-molecule chemical probe **SGC-iMLLT** for AF9/ENL YEATS domains was recently discovered. The complex crystal structure revealed that **SGC-iMLLT** displaces the natural substrate of the ENL YEATS domain [22]. A subsequent peptide displacement assay identified a dual submicromolar inhibitor of ENL and AF9 with a pharmacophore similar to that of **SGC-iMLLT** [23]. A cellular thermal shift assay was also utilized to discover sCGT990, a small-molecule inhibitor of the ENL/AF9 YEATS domain with an affinity of 14 μM. Interestingly, this compound represents a chemotype different from that of **SGC-iMLLT** [24]. In addition, peptide-based inhibitors of ENL YEATS domains have been developed with submicromolar affinities to the ENL/AF9 YEATS domain [25]. Conformationally preorganized cyclopeptides have also been revealed to target AF9 YEATS with 38-fold selectivity over ENL [26]. Although these peptides and small-molecule inhibitors interact with AF9/ENL in living cells, it is frustrating that these inhibitors in all cases have failed to suppress the proliferation of tumor cells and thus cannot recapitulate the phenotype induced by the depletion of the ENL gene [19,20]. Most recently, a new inhibitor **7** of ENL displayed selectivity over all other human YEATS domains and on-target inhibition of MLL-rearranged leukemia cell lines [27]. Additionally, **7** suppressed two ENL target genes’ expression, including MYC and HOXA9 in MOLM13 cells. Furthermore, small-molecule inhibitors targeting the AHD domain of ENL can suppress malignant gene expression and tumor growth in MLL-leukemia [28]. Interestingly, genome-scale CRISPR–Cas9 screens assessing the proliferation of 324 human cancer cell lines demonstrated that knockout of the ENL gene was effective in only one cell line [29]. Thus, these results lead to confusion over whether AF9/ENL YEATS domains can be used for therapeutic indications.

To clarify this issue, we aimed to discover inhibitors with new chemotypes and evaluate their antiproliferative capability in AF9-sensitive cells. Herein, we identified four small-molecule hits against the AF9 YEATS domain using NMR fragment-based screening [30,31]. The complex crystal structure revealed the key interactions between one of these hits and the AF9 YEATS domain. Derivatives of this hit with enhanced affinities to the AF9 YEATS domain were hence designed and synthesized. The complex structure of the hydrophobic interaction between the phenyl substituent of compound **10** and aromatic residues of the AF9 YEATS domain was delineated. Interestingly, AF9/ENL inhibitors preferentially suppressed the proliferation of gastric HGC-27 cells, consistent with the phenotype in genome-scale CRISPR studies. Our work paves the way for potent inhibitors of the AF9 YEATS domain for the treatment of AF9-sensitive cancers.

## 2. Results

**Figure 1:**
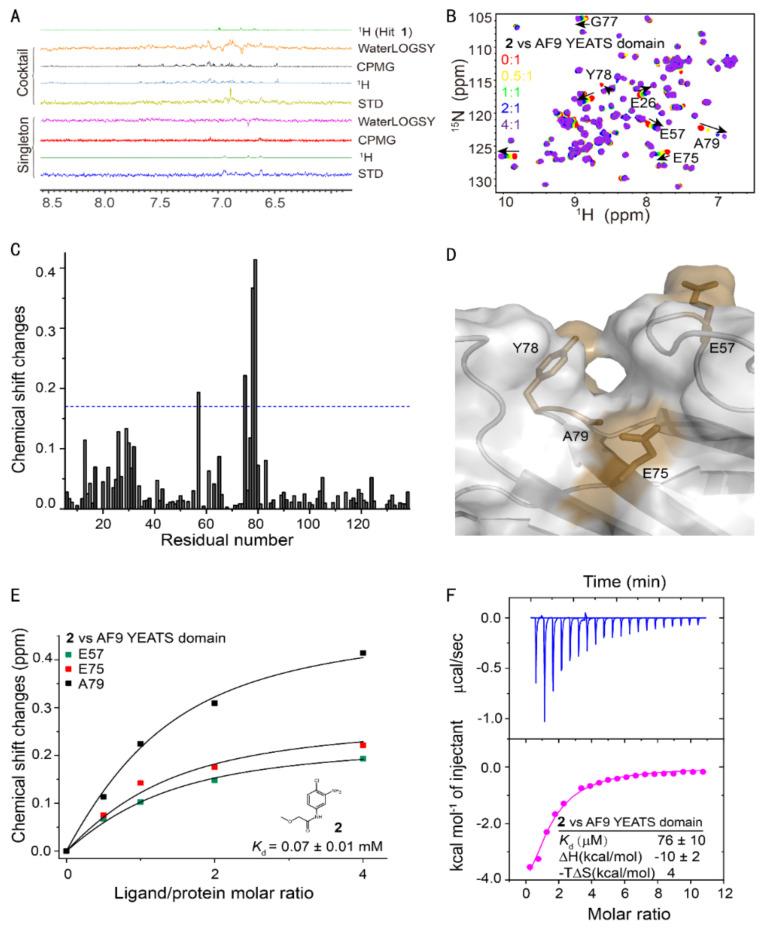
Fragment-based screening against the AF9 YEATS domain. (**A**) NMR fragment-based screening spectra for a typical cocktail and the identified hits of AF9 YEATS in a phosphate buffer. The reference ^1^H spectrum was acquired in 50% H_2_O/50% D_2_O. (**B**) The chemical shift perturbations of the ^15^N-labeled AF9 YEATS induced by hit **2**. The ligand/protein molar ratios are annotated. (**C**) The residue-by-residue chemical shift changes of the AF9 YEATS domain at a 4-fold excess of hit **2**. The dashed line represents two standard deviations above the average of the chemical shift perturbations. (**D**) The binding topology of **2** mapped on the surface of AF9 YEATS (PDB code: 4TMP). Residues of significant chemical shift changes are colored yellow. (**E**) The binding affinity of **2** derived from best fitting of the dose-dependent chemical shift changes of AF9 YEATS. The *K*_d_ value with a fitting error is annotated. (**F**) The binding enthalpy and affinity of AF9 YEATS and **2** determined by isothermal titration calorimetry, assuming a 1:1 binding mode.

Using our fragment-based lead discovery platform [32,33], we screened 89 cocktails with 10 compounds each against the AF9 YEATS domain using NMR ligand-observed spectra, i.e., Saturation Transfer Difference (STD), WaterLOGSY, and Carr–Purcell–Meiboom–Gill (CPMG) [34,35,36] (Figure 1A). In principle, only compounds that bind will show signals in the STD spectrum. Accordingly, WaterLOGSY and CPMG spectra will show inverted or remarkably reduced intensity relative to that of the nonbinding compounds. This primary screening identified 28 cocktails with binding signatures. The cocktail was then deconvoluted through screening of feasible hits as a singleton.

To further validate the results of ligand-observed fragment-based screening, we titrated these hits with the ^15^N-labeled AF9 YEATS domain (Figure 1B). The backbone chemical shift assignment was retrieved from the literature [7]. Four hits demonstrated significant chemical shift perturbations (CSPs) of at least two residues. The residue-by-residue CSPs suggested that hit **2** bound to a pocket proximal to residues E57, E75, Y78, and A79 (Figure 1C). These residues were then mapped to the surface of the crystal structure of the AF9 YEATS domain in complex with H3K9ac [10] (PDB code: 4TMP), which indicated the binding of these hits to the same pocket for acyl-lysine recognition (Figure 1D). The binding affinity of hit **2** was then determined by best fitting the dose-dependent CSPs of these residues (Figure 1E) at 0.07 ± 0.01 mM (fitting error was used henceforth unless specifically annotated). The affinity of hit **2** was further validated using isothermal titration calorimetry [37] (ITC) (Figure 1F). The binding affinity of the other hits was determined using the same approach (Table 1). These four hits probably bound to the same pocket since they shared the same pharmacophore.

**Figure 2:**
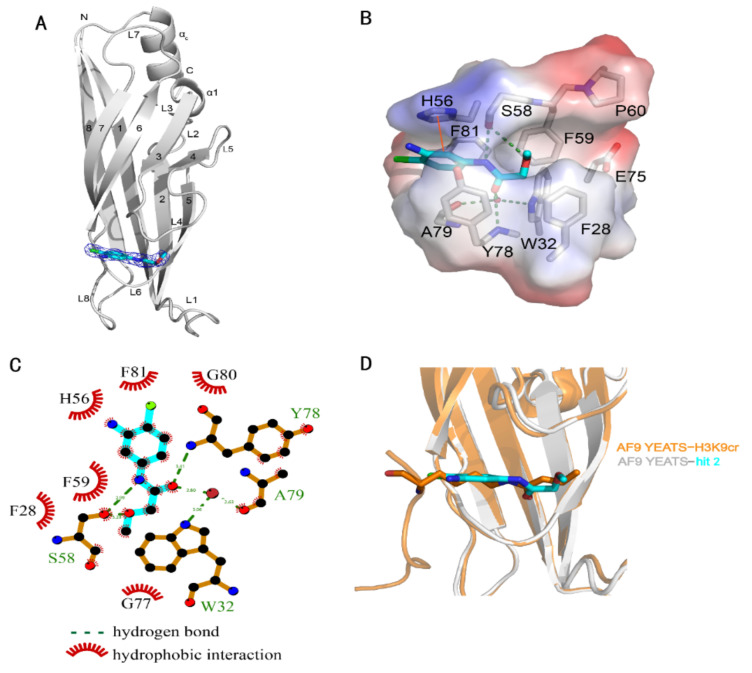
Crystal structure of the AF9 YEATS domain in complex with hit **2**. (**A**) Ribbon diagram of the structure of AF9 YEATS in complex with hit **2** (carbon atoms in cyan). The 2Fo–Fc electron density map of **2** was contoured at 1σ. (**B**) Detailed interactions between **2** and proximal residues of AF9 YEATS with hydrogen bonds (green dashed line) and π-π interactions (orange solid line) are delineated. (**C**) LIGPLOT program illustrating the contacts between AF9 YEATS domain and **2**. (**D**) Superimposition of the crystal structures of the AF9 YEATS in complex with **2** or crotonylated H3K9 (PDB code: 5HJB).

The crystal structure of the AF9 YEATS domain in complex with hit **2** was hence solved to depict their interaction mode (Table 2). The AF9 YEATS domain adopted an immunoglobin fold that consisted of a two-layer β sandwich between eight antiparallel β strands [7] (Figure 2A). The highly conserved residues F28, H56, S58, F59, Y78, and F81 together formed a serine-lined aromatic cage for acyl-lysine recognition, where hit **2** was embedded with a clearly visible electron density. The benzyl moiety of hit **2** interacted with H56 through π-π stacking. The amide nitrogen and ether oxygen of hit **2** participated in the formation of hydrogen bonds with the side-chain hydroxyl of S58, while the carbonyl group of hit **2** formed a hydrogen bond with the backbone amide of Y78 (Figure 2B). Furthermore, this carbonyl group also participated in the water-mediated hydrogen bonds with W32 and A79. Detailed hydrogen bonds and hydrophobic interactions were depicted using LIGPLOT (Figure 2C).

The structure of AF9 YEATS in complex with hit **2** indicated the feasibility to improve ligand potency. The substitution of aniline, which did not directly interact with AF9, by hydrogen or hydroxyl (compounds **5** and **6**) did not significantly alter the affinity (Table 1). Furthermore, superimposition of the crystal structures of AF9 YEATS in complex with hit **2** or H3K9cr peptide [6] (Figure 2D) suggested that the chlorine group of hit **2** may be substituted. However, compounds **7**–**9** exhibited weakened affinities, indicating that these compounds may not mimic the binding mode of H3K9cr well, possibly limited by the geometric restraints. As the crystal structure also revealed that the methoxyl group of hit **2** pointed toward the unoccupied groove surrounded by residues F28, P60, and E75 (Figure 2B), we substituted the methyl group with a benzyl or phenyl group (compounds **10** and **11**). Compound **10** achieved an approximately two-fold enhancement of the affinity for the AF9 YEATS domain.

**Figure 3:**
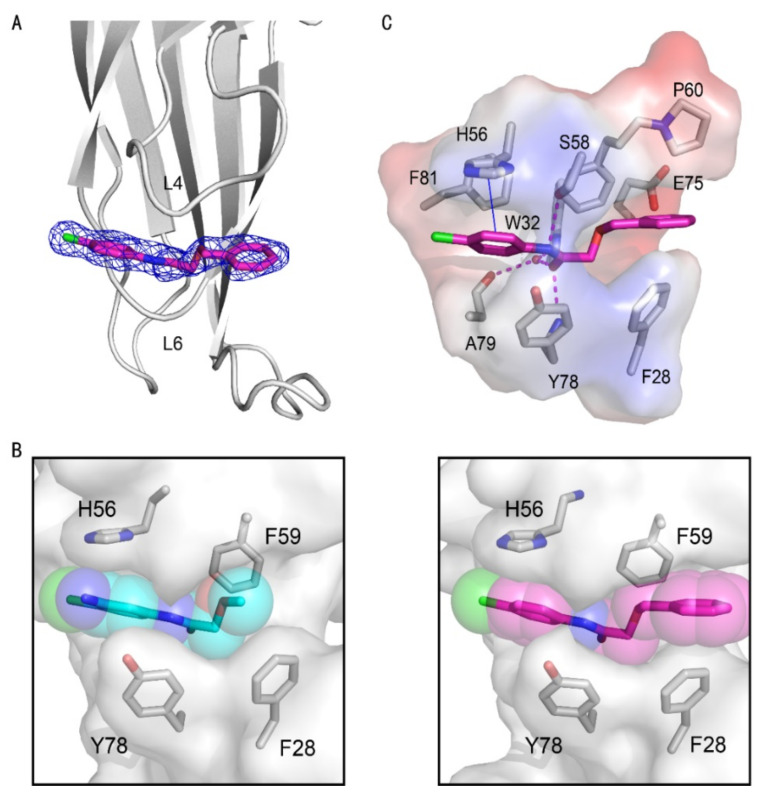
Crystal structures of the AF9 YEATS domain in complex with compound **10**. (**A**) Cartoon representation of the AF9 YEATS domain in complex with compound **10** (carbon atom in pink), whose 2Fo–Fc electron density map was contoured at 1σ (navy mesh). (**B**) Comparison of cavity encapsulation of compounds **2** and **10** that were shown as spheres. (**C**) Detailed interactions between compound **10** and AF9 YEATS with hydrogen bonds (pink dashed line) and π-π interaction interactions are highlighted (blue solid line).

We hence solved the crystal structure of the AF9 YEATS domain in complex with compound **10** with clear electron density, diffracted at 1.8 Å (Table 2, Figure 3A). In general, compounds **2** and **10** demonstrated a conserved pose and thus shared a similar interaction pattern. However, compound **10** bound deeper along the same aromatic groove and thus had a more intimate cavity encapsulation than compound **2** did (Figure 3B). Therefore, the benzyl substituent of compound **10** had additional interactions with residues P60 and F28 (Figure 3C) for enhanced affinity to AF9 YEATS.

**Figure 4:**
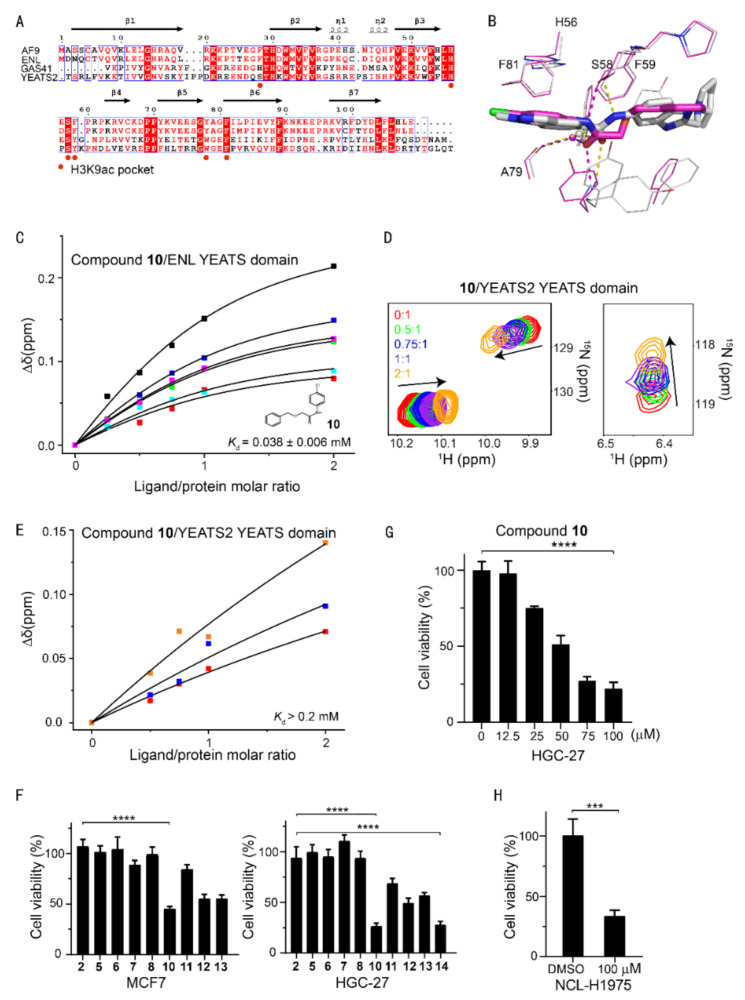
Selectivity analysis of AF9 inhibitors among human YEATS domains and antiproliferation activities. (**A**) Sequence alignment of human YEATS domains. Conserved residues are highlighted, with residues interacting with H3K9ac annotated. (**B**) Superimposition of AF9 and ENL YEATS domain structures in complex with **10** (carbon atom in magenta) and **SGC-iMLLT** (PDB code: 6HT1), respectively. The hydrogen bonds are delineated by dashed lines. (**C**) The binding affinity of **10** determined from the dose-dependent chemical shift perturbations of ENL YEATS. The *K*_d_ value with the fitting error is annotated. (**D**) Chemical shift perturbations of the ^15^N-labeled YEATS2 YEATS domain induced by **10**. (**E**) The binding affinity of **10** estimated from best fitting of the dose-dependent chemical shift perturbations of the YEATS2 YEATS domain. (**F**) Viability of MCF7 and AF9-sensitive HGC-27 cells upon treatment with inhibitors at a single dose of 100 μM. The values represent mean ± SEM of 8 independent experiments (**** *p* < 0.0001, two-tailed unpaired Student‘s *t*-test). (**G**) Cell growth inhibition of compound **10** at various concentrations in the HGC-27 cell line. The values represent mean ± SEM of 4 independent experiments. (**H**) Viability of NCL-H1975 cells upon treatment with **10** at a single dose of 100 μM (*** *p* < 0.001 two-tailed unpaired Student‘s *t*-test).

Sequence alignment of the AF9/ENL YEATS domains suggested a conserved aromatic cage in AF9 and ENL (Figure 4A). It is, therefore, difficult to achieve a high selectivity between AF9 and ENL YEATS domains, as underscored by the similar affinity of compound **10** to both AF9 and ENL (38 ± 6 μM) determined by CSPs (Figure 4C), even though compound **10** represented a new chemotype with a binding mode distinct from that of **SGC-iMLLT** (Figure 4B). Conversely, compound **10** only slightly disturbed the chemical shift of YEATS2 (Figure 4D), thus showing very weak binding affinity (Figure 4E). Therefore, compound **10** was a dual inhibitor of AF9/ENL YEATS domains.

A series of derivatives of **10** were therefore designed and synthesized (compounds **12**–**14**), which were aimed at inducing more interactions with residues proximal to the aromatic cage. Difluorinated compound **14** gained an approximately 30% affinity enhancement relative to that of **10,** indicating the fluorine atoms contributed additional interactions with residue Y78/F81 of AF9. We further assessed the cellular activity of these inhibitors in cancer cells. Although knockout of the *ENL* gene has been shown to suppress the proliferation of acute myeloid leukemia [19,20], this phenotype was not recapitulated by treatment with known ENL inhibitors [22]. Thus, this raises doubts about the discovery of potent AF9/ENL YEATS domain inhibitors. It is also worth noting that cellular activities may be modulated by many factors, e.g., affinity, cellular permeability, metabolic stability, etc. However, cancer therapeutic targets have been recently prioritized using genome-scale CRISPR–Cas9 screens in 324 human cancer cell lines, which revealed that knockout of the *AF9* or *ENL* gene suppressed the proliferation of nine or one cancer cell lines, respectively [29]. Specifically, the knockout of AF9 gene suppressed the proliferation of HGC-27 but not MCF7 cells. Meanwhile, the ENL YEATS domain inhibitor, **SGC-iMLLT**, exhibited weak potency in MCF7, and almost no detectable activities over the rest of the 59 cell lines. Interestingly, the expression level of AF9 in MCF7 cells is significantly higher than that of other YEATS proteins, while the expression levels of YEATS family proteins are similar in gastric cancer cell lines (https://www.proteinatlas.org/ accessed on 28 January 2022). Compound **10** had weak potency in MCF7 cells using a Cell Counting Kit-8 (CCK-8) assay, but suppressed the proliferation of AF9-sensitive HGC-27 cells more effectively in a dose-dependent manner (Figure 4F, 4G), which is consistent with the CRISPR result [29]. Accordingly, compound **10** demonstrated inhibitory effects in another AF9-sensitive cell line NCL-H1975 (Figure 4H). We thus speculate that more potent inhibitors of the AF9/ENL YEATS domain may demonstrate higher cellular activity. The affinity of the (R)-or (S)-enantiomer of **SGC-iMLLT** to the ENL YEATS domain has previously been determined to be 0.83 and 0.13 μM, respectively. We hence synthesized the racemate of **SGC-iMLLT** due to the limited material availability. This compound suppressed the proliferation of HGC-27 cells at an IC_50_ of 19 μM using the CCK8 assay. Taken together, our data underpins the campaign to uncover more potent inhibitors of AF9 YEATS for the treatment of AF9-sensitive cancers.

## 3. Conclusions

The AF9/ENL YEATS domains recognize acylated histones to recruit the super elongation complex and, in turn, DOT1L on active chromatin. Dysregulation of AF9/ENL thus correlates with the onset and progression of diseases, particularly cancers. Small-molecule inhibitors and peptide-mimics have recently been discovered to block the recognition of acyl-lysines by the ENL YEATS domain. However, the ENL inhibitor **SGC-iMLLT** and its analogs failed to suppress the proliferation of cancer cells, while compound **7** with a new chemotype did [27]. The large-scale prioritization of cancer targets nominated 9 out of 324 cell lines as AF9-sensitive cell lines, thus reigniting the campaign toward the discovery of AF9 YEATS inhibitors. We identified four hits of the AF9 YEATS domain from fragment-based screening against a library of 890 compounds. Inhibitors harboring the new chemotypes were then uncovered guided by complex crystal structures, which revealed a binding mode to form an extra hydrogen bond in comparison with **SGC-iMLLT**. These compounds displayed antiproliferation activities in the AF9-sensitive HGC-27 cells. Taken into account of the rotatable bonds of the linker between the two aromatic rings, further optimization of a new lead compound to cyclize the linker is currently on the way. Furthermore, the complex crystal structures reveal feasibility to refine substitutions on the aromatic groups. In general, our data underpins the further pursuit of a more potent candidate, which may probe downstream cellular functions, e.g., the expression of MYC and HOXA9 genes, to validate AF9 inhibition as anticancer treatment. 

## 4. Materials and Methods

### 4.1. Cloning, Protein Expression, and Purification

DNA fragment encoding AF9 YEATS domain (residues 1–138) was amplified and cloned into the pET28a vector (GE Healthcare, Chicago, IL, USA). The construct was then transformed into Escherichia coli BL21 (DE3) by heat shock. The protein expression was induced at A600 = 0.8–1.0 using 0.3 mM Isopropyl β-d-1-thiogalactoside. After incubating at 25 °C for 20 h, the cell pellets were harvested by centrifugation and resuspended in a binding buffer of 20 mM HEPES, 0.5 M NaCl at pH 7.4. The cells were then lysed by a high-pressure cracker and centrifuged at 13,000× *g* for 30 min. The supernatant was purified on a HisTrap nickel column (GE Healthcare) and a gel-filtration column (Superdex 75 30/100 GL) in 20 mM Tris, 150 mM NaCl, 2 mM DTT, 1 mM EDTA at pH 7.4. Pure fractions as analyzed by SDS-PAGE were concentrated. Uniformly 15N-labeled protein was prepared from cells grown in LR medium supplemented with 15NH4Cl. The purified protein was dialyzed into PBS buffer for following NMR experiments.

### 4.2. Ligand-Observed Fragment Screening

The NMR ligand-observed fragment screening experiments were carried out at 25 °C using an Agilent 700 MHz spectrometer equipped with a 96-well autosampler and a cryoprobe. The ligand-based NMR spectra, i.e., saturation transfer difference (STD) [34], WaterLOGSY [35], CPMG [38], were acquired for 89 samples, each containing 10 μM of AF9 YEATS domain in PBS buffer and 10 fragment compounds at 400 μM each. The acquisition parameters of these NMR experiments were the same as previously published [39]. A follow-up screening was performed using the same NMR parameters against each individual hit. The NMR spectra were automatically processed and visualized using home-made ACD/Labs scripts.

### 4.3. NMR Chemical Shift Perturbation

The ^15^N-labeled AF9 YEATS domain was concentrated to 0.1 mM in phosphate buffered saline at pH 6.8. The HSQC spectra were acquired on the Agilent 500 MHz spectrometer at a ligand/protein molar ratio varying from 0 to 8.0. The dissociation constant *K*_d_ was best-fitted using the following equation assuming a 1:1 binding mode [40],

$$\Delta \delta_{obs}=\Delta \delta_{\max}\left[P_{{}_{t}}+L_{t}+K_{d}-\sqrt{{\left(P_{t}+L_{t}+K_{d}\right)}^{2}-4P_{t}L_{t}}\right]/2P_{t}$$

where *P_t_* and *L_t_* represented the total concentrations of the protein and the ligand, respectively, and the Δ*δ_obs_* denoted the observed chemical shift changes relative to the free-form protein. Δ*δ_obs_* was defined as $\sqrt{1/2\left[\delta_{H}^{2}+0.04\delta_{N}^{2}\right]}$, where *δ*_H_ and *δ*_N_ were the chemical shift changes in the ^1^H and ^15^N dimension, respectively. The maximum of the chemical shift perturbations Δ*δ_max_* and the dissociation constant *K*_d_ were best-fitted from the dose-dependent chemical shift changes

### 4.4. Isothermal Titration Calorimetry

All ITC titrations were carried out on a MicroCal ITC200 calorimeter (Malvern Instruments Ltd., Malvern, UK) at 298K. The AF9 YEATS domain protein was dialyzed to 20 mM Tris buffer containing 150 mM NaCl at pH 7.4. The concentration of the protein was determined by UV-Vis spectroscopy. The compounds were dried and dissolved in the same buffer. The concentrations of compounds were determined using quantitative NMR30. The ITC data were analyzed using Origin 2019 and best-fitted to the one-site binding model to determine the enthalpies and affinity constants. The binding entropies were calculated using the Gibbs free energy equation.

### 4.5. Crystallization, Data collection

The protein was concentrated to 8–10 mg/mL in a buffer containing 20 mM Tris, 150 mM NaCl, 1mM EDTA, 2 mM DTT at pH 7.4, and then incubated with the compounds at 2:1 molar ratio overnight at 8 °C. The complex crystals of AF9 YEATS domain and compounds were generated by mixing 1 μL supernatant with 1 μL reservoir solution containing 0.2 M trimethylamine N-oxide dehydrate, 0.1 M Tris, 20% *w/v* PEGMME 2000, pH 8.5 using the sitting-drop vapor diffusion method at 20 °C. Crystals were flash-frozen in liquid nitrogen under cryoprotectant conditions (reservoir solution supplemented with 25% glycerol). The crystallographic data were collected at beamline BL18U/BL19U at the Shanghai Synchrotron Radiation Facility (SSRF).

### 4.6. Structure Determination and Refinement

All crystallographic data sets were indexed, integrated, and scaled by HKL2000 suite [41]. The ligand was prepared using the ProDrg program of CCP4 [42], and the complex structure was determined using Phaser MR in CCP4 with the complex structure (PDB ID: 4TMP) [10] as the search model. The initial model was built with COOT [43] and further refined using Refmac5 and PHENIX [44]. All the figures were prepared by PyMOL (DeLano Scientific LLC, Palo Alto, CA, USA).

### 4.7. Cell Viability Assay

Human MCF7 and HGC-27 cell lines were cultured at 37 °C in a humidified 5% CO2 atmosphere in DMEM (Gibco, Stony Plain, AB, Canada) medium supplemented with 10% fetal bovine serum (FBS) (Gibco) and 1 × penicillin/streptomycin (Gibco). MCF7 and HGC-27 cells were seeded in 96-well plates with approximately 10^4^ cells in 100 μL of the medium and treated with the following conditions: fresh culture medium with DMSO (control) or fresh culture medium with inhibitors at a concentration of 100 μM. Cell viability was measured using a Cell Counting Kit-8 (Purchased from TargetMol, Boston, MA, USA) according to manufacturer’s instructions [45]. The absorbance was read at 450 nm with a microplate reader (CLARIOstar, BMG LABTECH, Ortenberg, Germany). Cell viability was calculated as the ratio of experimental group absorbance vs. control group absorbance.

### 4.8. Chemical Synthesis and Characterization

Compound **5**/**6**


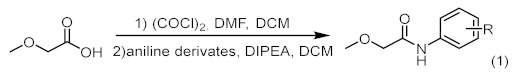


Dissolved methoxyacetic acid (1 eq.) in the solvent dichloromethane (DCM) and placed the reaction vessel in an ice bath at 0 °C. N, N-dimethylformamide (DMF) (0.05 equivalent, eq.) was added dropwise thereto. Under stirring conditions, oxalyl chloride (2 eq.) was slowly added dropwise. After the addition was complete, the system reacted at room temperature for half an hour. Afterwards, the solvent was spin-dried for subsequent use. The aniline derivative (1.2 eq.) was dissolved in DCM, N, N-diisopropylethylamine (DIPEA) (1.3 eq.) was added to the reaction system, and the reaction was stirred at 0 °C. The prepared acid chloride dichloromethane solution was slowly added dropwise to the system. After the addition was completed, the reaction was stirred overnight at room temperature. Afterwards, the reaction system was quenched by adding water, stirred, and extracted with dichloromethane three times. The organic phases were combined, washed with brine, dried over anhydrous sodium sulfate, filtered, the filtrate was spin-dried, and the crude product was separated by silica gel column chromatography to obtain amide compounds **5** and **6**.

Compound **7**


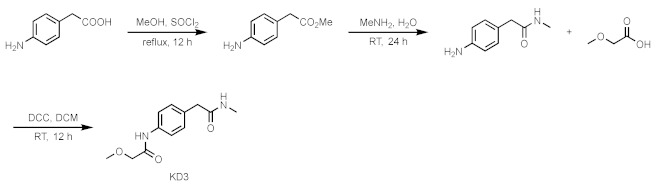


4-aminophenylacetic acid (0.500 g, 3.65 mmol) was dissolved in methanol (20 mL), and SOCl_2_ was added dropwise. Heated to reflux for one hour and then removed the solvent in a vacuum. The crude product was separated by column chromatography to obtain the desired white solid compound 4-amino phenylacetate methyl ester. It was dissolved in 40% methylamine (8 mL) aqueous solution and stirred at room temperature for 24 h. It was separated by column chromatography to obtain 4-amino-N-methylbezeneacetamide as a pale yellow oily product. Subsequently, the product was dissolved in DCM (5 mL), and placed at 10 °C. Then, DCC (216 mg 1.05 mmol) was added to the reaction system. Then, methoxyacetic acid was dissolved in DCM, added dropwise, stirred at room temperature for 12 h, and the organic solvent was removed in a vacuum to obtain the product compound **7** by column chromatography.

Compound **8**/**9**


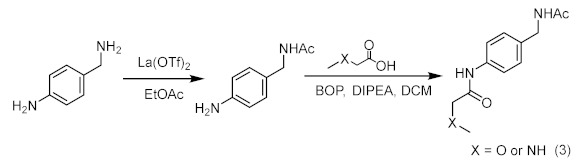


Added lanthanum trifluoromethanesulfonate (0.05 eq.) and 4-aminobenzylamine (1.2 eq.) into a two-necked flask equipped with a stir bar. After replacing with argon three times, added ethyl acetate at room temperature (1.0 eq.) and reacted at 50 °C for 24 h. After the reaction, the crude mixture was diluted with dichloromethane and purified by silica gel flash column chromatography to obtain the intermediate product of amide. At room temperature, the intermediate product (1.0 eq.) and acid (1.0 eq.) were dissolved in anhydrous DCM. DIPEA (3.0 eq.) was added, and then a dichloromethane solution of Carter condensing agent (BOP) (1.0 eq.) was added, and the mixture was stirred at room temperature for 12 h. Afterwards, the solvent was spin-dried and purified by column chromatography on a silica gel column to obtain the final products **8** and **9**.

Compounds **10**–**14**


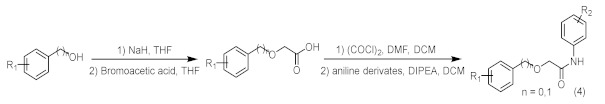


Added sodium hydride (NaH) (2.2 eq.) to a two-necked flask equipped with a stir bar, replaced with argon three times, added tetrahydrofuran (THF) at 0 °C to make a solution, and then added phenol or benzyl alcohol derivatives (2.2 eq.) to the reaction system, stirred at 0 °C for 30 min. After the reaction was completed, a solution of bromoacetic acid (1.0 eq.) in tetrahydrofuran was added to the system and reacted for 12 h under reflux conditions. After the reaction was completed, added water to quench, washed with ethyl acetate three times, and then added 1 M dilute hydrochloric acid solution to the aqueous phase dropwise. After adjusting the pH value near 3, extracted three times by ethyl acetate, mixed the organic phases, brine washed, dried with anhydrous sodium sulfate, spin-dried to obtain the acid intermediate, which could be used directly. The acid (1 eq.) was dissolved in the solvent DCM, the reaction vessel was placed in an ice bath at 0 °C, and DMF (0.05 eq.) was added dropwise. Under stirring conditions, oxalyl chloride (2 eq.) was slowly added dropwise. After the addition was complete, the system reacted at room temperature for half an hour. Then, the solvent was spin-dried and set aside. The aniline derivative (1.2 eq.) was dissolved in DCM, DIPEA (1.3 eq.) was added to the reaction system, and the reaction was stirred at 0 °C. The prepared acid chloride dichloromethane solution was slowly added dropwise to the system. After the dropwise addition was completed, the reaction was stirred overnight at room temperature. After the reaction was completed, the reaction system was quenched by adding water, stirred, and extracted with dichloromethane three times. The organic phases were mixed, washed with brine, dried over anhydrous sodium sulfate, filtered, the filtrate was spin-dried, and the crude product was separated by silica gel column chromatography to obtain amide compounds **10**–**14**.

**SGC-iMLLT** racemate

Synthesized according to general procedure B to produce **SGC-iMLLT** racemate [22]. 

Compound **5**: ^1^H NMR (500 MHz, Chloroform-d) δ 8.28 (s, 1H), 7.53 (d, J = 8.8 Hz, 2H), 7.29 (d, J = 8.8 Hz, 2H), 4.00 (s, 2H), 3.49 (s, 3H). ^13^C NMR (126 MHz, CDCl3) δ = 167.7, 135.8, 129.5, 129.1, 121.1, 72.1, 59.4.

Compound **6**: ^1^H NMR (500 MHz, DMSO-*d_6_*) δ 10.2 (s, 1H), 9.8 (s, 1H), 7.6 (d, *J* = 2.4 Hz, 1H), 7.2 (d, *J* = 8.6 Hz, 1H), 7.0 (dd, *J* = 8.6, 2.4 Hz, 1H), 4.0 (s, 2H), 3.4 (s, 3H). ^13^C NMR (126 MHz, DMSO) δ 168.1, 152.9, 138.2, 129.5, 114.0, 111.6, 107.9, 71.7, 58.6.

Compound **7**: ^1^H NMR (500 MHz, DMSO-*d_6_*) δ 9.7 (s, 1H), 7.9 (s, 1H), 7.6 (d, *J* = 8.6 Hz, 2H), 7.2 (d, *J* = 8.6 Hz, 2H), 3.9 (s, 3H), 2.6 (s, 2H), 2.5 (d, *J* = 4.3 Hz, 3H), 2.4 (s, 2H). ^13^C NMR (126 MHz, DMSO) δ 170.6, 167.9, 136.7, 131.6, 129.1, 119.7, 71.7, 58.6, 41.8, 25.6.

Compound **8**: ^1^H NMR (500 MHz, DMSO-*d_6_*) δ 9.7 (s, 1H), 8.3 (t, *J* = 6.0 Hz, 1H), 7.6 (d, *J* = 8.5 Hz, 2H), 7.2 (d, *J* = 8.4 Hz, 2H), 4.2 (d, *J* = 5.9 Hz, 2H), 4.0 (s, 2H), 3.4 (s, 3H), 1.9 (s, 3H). ^13^C NMR (126 MHz, DMSO) δ 169.1, 167.9, 137.1, 134.7, 127.6, 119.7, 71.7, 58.6, 41.7, 22.6.

Compound **10**: ^1^H NMR (400 MHz, Chloroform-d) δ 8.33 (s, 1H), 7.50 (d, J = 8.2 Hz, 2H), 7.42–7.33 (m, 5H), 7.27 (d, J = 8.9 Hz, 2H), 4.64 (s, 2H), 4.08 (s, 2H). ^13^C NMR (101 MHz, CDCl3) δ = 167.7, 136.6, 135.9, 129.6, 129.2, 128.9, 128.6, 128.2, 121.2, 74.0, 69.7.

Compound **11**: ^1^H NMR (400 MHz, Chloroform-d) δ 8.34 (s, 1H), 7.53 (d, J = 8.8 Hz, 2H), 7.38(d, J = 8.8 Hz, 2H), 7.30 (t, 2H), 7.05 (t, J = 7.4 Hz, 1H), 6.96 (d, J = 8.4 Hz, 2H), 4.56 (s, 2H). ^13^C NMR (101 MHz, CDCl3) δ = 157.0, 135.5, 130.0, 129.9, 129.2, 122.6, 121.5, 114.9, 67.6.

Compound **12**: ^1^H NMR (500 MHz, DMSO-*d_6_*) δ 9.9 (s, 1H), 7.7 (d, *J* = 8.6 Hz, 2H), 7.5 (dd, *J* = 8.4, 5.6 Hz, 2H), 7.4 (d, *J* = 8.5 Hz, 2H), 7.2 (t, *J* = 8.8 Hz, 2H), 4.6 (s, 2H), 4.1 (s, 2H). ^13^C NMR (126 MHz, DMSO) δ 168.2, 161.7 (d, *J* = 243.5 Hz), 137.4, 133.9 (d, *J* = 3.4 Hz), 130.0 (d, *J* = 8.2 Hz), 128.5, 127.2, 121.3, 115.1 (d, *J* = 21.2 Hz), 71.7, 69.4.

Compound **13**: ^1^H NMR (500 MHz, DMSO-*d_6_*) δ 9.9 (s, 1H), 9.7 (s, 1H), 7.7 (d, *J* = 8.6 Hz, 2H), 7.4 (d, *J* = 8.6 Hz, 2H), 7.3 (d, *J* = 7.5 Hz, 1H), 7.2 (t, *J* = 7.7 Hz, 1H), 6.9 (d, *J* = 8.0 Hz, 1H), 6.8 (t, *J* = 7.4 Hz, 1H), 4.6 (s, 2H), 4.1 (s, 2H). ^13^C NMR (126 MHz, DMSO) δ 169.2, 155.9, 137.7, 130.3, 129.6, 129.0, 127.7, 124.0, 121.7, 119.3, 115.6, 69.7, 69.0.

Compound **14**: ^1^H NMR (500 MHz, DMSO-*d_6_*) δ 9.7 (s, 1H), 7.5–7.4 (m, 4H), 7.4 (t, *J* = 7.4 Hz, 2H), 7.3 (t, *J* = 7.2 Hz, 1H), 4.6 (s, 2H), 4.2 (s, 2H). ^13^C NMR (126 MHz, DMSO) δ 168.6, 157.9 (dd, *J* = 251.2, 6.6 Hz), 137.5, 131.7 (t, *J* = 13.1 Hz), 128.3, 127.9, 127.7, 113.7 (t, *J* = 17.2 Hz), 113.0 (dd, *J* = 21.2, 5.8 Hz), 72.5, 69.0.

## Figures and Tables

**Table 1:** Structure and affinities of fragment-derived inhibitors of the AF9 YEATS domain.

ID	Structure	*K_d_* (mM) ^a^	LE	ID	Structure	*K_d_* (mM)	LE
**1**	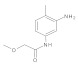	0.137 ± 0.024 ^b^	0.38	**8**	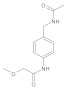	0.31 ± 0.004	0.36
**2**	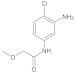	0.07 ± 0.01	0.41	**9**	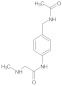	0.66 ± 0.07	0.26
**3**	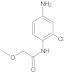	0.10 ± 0.01	0.39	**10**	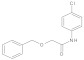	0.038 ± 0.006	0.42
**4**	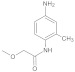	0.44 ± 0.10	0.33	**11**	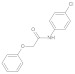	0.088 ± 0.03	0.31
**5**	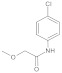	0.08 ± 0.06	0.43	**12**	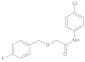	0.087 ± 0.011	0.28
**6**	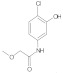	0.061 ± 0.004	0.41	**13**	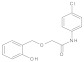	0.07 ± 0.009	0.28
**7**	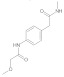	0.27 ± 0.02	0.29	**14**	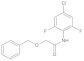	0.026 ± 0.004	0.30

**^a^***K*_d_ values were determined from the dose-dependent chemical shift perturbations. **^b^** Fitting error.

**Table 2:** X-ray crystallography data collection and refinement statistics for the AF9-inhibitor complex.

PDB ID	7VKH	7VKG
**Data Collection**		
Space group	*P*1	*P*2_1_
Cell dimensions		
a, b, c (Å)	31.652, 41.426, 59.725	41.381, 31.553, 89.378
α, β, γ (°)	102, 90.87, 90.357	90, 102.04, 90
Wavelength (Å)	0.9774	0.9785
Resolution (Å)	40.00–2.25 (2.29–2.25) *	40.00–1.83 (1.86–1.83) *
Completeness (%)	97.6 (93.6)	99.1 (97.4)
Redundancy	3.3 (2.5)	6.6 (5.9)
*R*_sym_ or *R*_merge_ (%)	11.1 (38.1)	7.9 (65.2)
I/σI	9.26 (2.05)	20.44 (2.67)
**Refinement**		
No. reflections used/free	13,767/703	13,843/649
*R*_work_/*R*_free_ (%)	20.61/24.45	20.20/24.54
R.m.s.deviations		
Bond lengths (Å)	0.003	0.004
Bond angles (°)	0.669	0.767
B-factors (Å^2^)		
Protein	28.08	26.00
Ligand	33.16	31.26
Water	26.91	32.43
No. atoms		
Protein	2228	1116
Ligand	28	19
Water	35	61
Ramachandran plot		
Favored/allowed/outlier (%)	98.87/1.13/0	100/0/0

* Values in parentheses are for highest-resolution shell.

## Data Availability

Structure data are deposited in the Protein Data Bank with the accession code 7VKH and 7VKG.

## Abbreviations

YEATS	YAF9, ENL, AF9, TAF14, SAS5
STD	Saturation Transfer Difference
CPMG	Carr–Purcell–Meiboom–Gill
CSPs	Chemical shift perturbations
HSQC	Heteronuclear single-quantum coherence
NMR	Nuclear Magnetic Resonance
